# Prognostic nomograms to predict overall survival and cancer‐specific survival in patients with pelvic chondrosarcoma

**DOI:** 10.1002/cam4.2452

**Published:** 2019-07-29

**Authors:** Li Chen, Cheng Long, Jiaxin Liu, Xin Duan, Zhou Xiang

**Affiliations:** ^1^ Department of Orthopedics West China Hospital, Sichuan University Chengdu China

**Keywords:** cancer‐specific survival, chondrosarcoma, nomogram, overall survival, pelvis, SEER

## Abstract

**Background:**

The pelvis is the most common site of chondrosarcoma (CS), and the prognosis for patients with pelvic CS is worse than that for patients with CS in the extremities. However, clinicians have had few tools for estimating the likelihood of survival in patients with pelvic CS. Our aim was to develop nomograms to predict survival of patients with pelvic CS.

**Methods:**

Data from the Surveillance, Epidemiology, and End Results (SEER) database of patients with pelvic CS between 2004 and 2016 were retrieved for retrospective analysis. Univariate and multivariate Cox analyses were used to identify independent prognostic factors. On the basis of the results of the multivariate analyses, nomograms were constructed to predict the likelihood of 3‐ and 5‐year overall survival (OS) and cancer‐specific survival (CSS) of patients with pelvic CS. The concordance index (C‐index) and calibration curves were used to test the models.

**Results:**

In univariate and multivariate analyses of OS, sex, pathologic grade, tumor size, tumor stage, and surgery were identified as the independent risk factors. In univariate and multivariate analyses of CSS, pathologic grade, tumor size, tumor stage, and surgery were identified as the independent risk factors. These characteristics except surgery were integrated in the nomograms for predicting 3‐ and 5‐year OS and CSS, and the C‐indexes were 0.758 and 0.786, respectively.

**Conclusion:**

The nomograms precisely and individually predict OS and CSS of patients with pelvic CS and could aid in personalized prognostic evaluation and individualized clinical decision‐making.

## Graphical abstract

Independent prognostic factors associated with overall survival and cancer‐specific survival of pelvic chondrosarcoma were identified.This is the first time to develop and validate nomograms to predict prognosis in patients with pelvic chondrosarcoma, and it could be beneficial to clinicians in making personalized survival evaluation.

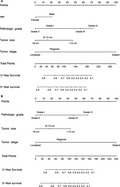

## 1 INTRODUCTION

Chondrosarcoma (CS) is a rare malignancy composed of cartilage‐producing cells that account for ~30% of all malignant bone tumors.^1^ It is the second most common primary malignant bone tumor after osteosarcoma.^2^ The annual incidence of CS is estimated to be 0.2/100 000 in Europe, and it typically affects adults older than 40 years.^3^ The pelvis is reported to be the most common primary site of CS, and patients with pelvic CS tend to have worse outcomes than those occurred in the extremities.^4^, ^5^ This tendency is presumably associated with the complex anatomy of the pelvis and the proximity of tumor to internal organs and neurovascular structures.^6^ However, due to the rarity of pelvic CS, few previous studies have focused on determining the prognosis of affected patients.^7^ Prognostic factors associated with survival are multivariate, and so no single factor can accurately predict outcomes of pelvic CS. Therefore, we sought to develop a prognostic model that was based on large samples, which can incorporate all prognostic factors to individually predict survival of patients with pelvic CS.

Nomograms, statistic‐based tools integrating all independent prognostic factors, have been widely applied to predict survival outcomes with precision for individual patients with cancer, including osteosarcoma, lung cancer, rectal cancer, and gastric cancer.^8^, ^9^, ^10^, ^11^ However, to our knowledge, the application of prognostic nomograms for pelvic CS has not been reported. The Surveillance, Epidemiology, and End Results (SEER) database released a large amount of clinical information about patients with pelvic CS that allowed prognostic analysis for pelvic CS. In this study, we used those data to identify independent prognostic factors affecting the overall survival (OS) and cancer‐specific survival (CSS) of patients with pelvic CS and to construct nomograms for visually predicting rates of 3‐ and 5‐year OS and CSS among patients with this disease.

## 2 PATIENTS AND METHODS

### 2.1 Ethics statement

This study was deemed exempt by the Ethics Committee of West China Hospital, Sichuan University (Chengdu, China), as it is based on the data extracted from the publicly available SEER database.

### 2.2 Data source and selection

We used SEER*Stat software version 8.3.5 (https://seer.cancer.gov/seerstat/) to obtain SEER data of about patients with pelvic CS who were diagnosed and treated between 2004 and 2016. This database annually updates the clinical cancer information from 18 regional cancer registries covering ~28% of the US population, including patients’ demographic characteristics, tumor characteristics, therapy details, and follow‐up records.^12^

**Figure 1:**
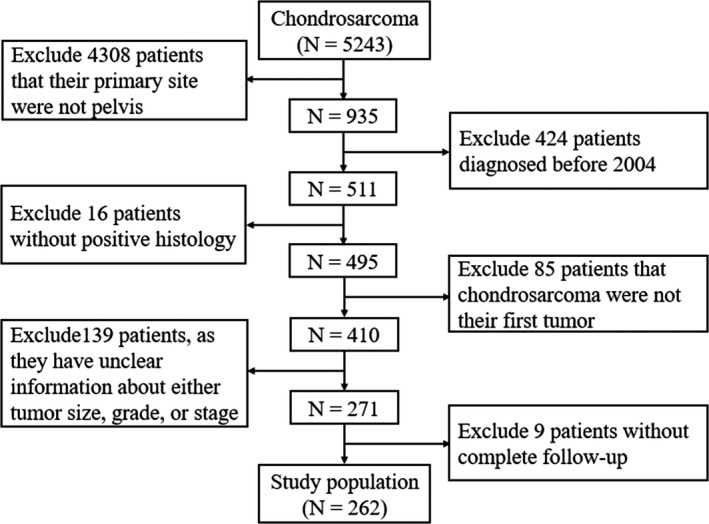
Flow diagram of selecting process in the Surveillance, Epidemiology, and End Results database

As shown in Figure 1, we selected specific cases if sufficient data were available from the database. The inclusion criteria were as follows: (a) diagnosed as 4.2‐CS (AYA site recode/WHO 2008) with the primary site limited to diagnosis code C41.4 (pelvic bones; *International Classification of Diseases,* 10th edition); (b) positive histological confirmation of CS; (c) completed follow‐up; (d) available information about months of survival and causes of death. Exclusion criteria were as follows: (a) tumor was not the patient's first cancer; (b) unknown tumor size, grade, or stage; and (c) unknown use of surgery, radiotherapy, or chemotherapy.

### 2.3 Study variables

Information about clinicopathological features included age at diagnosis, sex, race, histologic subtype, pathologic grade, tumor size (CS tumor size, 2004+), tumor stage (SEER historical stage A), treatment, vital status, and months of survival. The age at diagnosis was categorized as younger than 40, 40‐59, and 60 years and older.^13^ Race was categorized as White and others (Black, American Indian/Alaskan Native, Asian/Pacific Islander). The screened eligible cases were composed of three histologic subtypes based on the variable “ICD‐O‐3 Hist/behave:” conventional, myxoid, and dedifferentiated CS. Pathologic grade was classified according to four categories based on the variable “ICD‐O‐3 Grade:” Grades I, II, III, and IV. Tumor size was classified on the basis of the largest tumor diameter (<8, 8‐13, and >13 cm) according to the variable “CS tumor size (2004+).”^13^, ^14^ Tumor stage was categorized as localized, regional, and distant according to the variable “SEER historical stage A.” As described in the 2018 version of the *Summary Stage Manual* provided by SEER (https://seer.cancer.gov/tools/ssm/), localized tumors were defined as tumor confined to the pelvis or involvement of one to two pelvic segments without extraosseous extension. Regional tumors were defined as involvement of one to two pelvic segments with extraosseous extensions and without distant metastasis.

### 2.4 Statistical analysis

SPSS 24.0 (IBM Corp.) was used to evaluate the prognostic effect of each patient variable. We used the Kaplan‐Meier method to construct cumulative survival curves and the log‐rank test to compare them. OS and CSS were chosen as the two primary survival outcomes in this study. OS was defined as the period from diagnosis to death from any causes. CSS was defined as the period from diagnosis to death attributed to pelvic CS.

Cox proportional hazard models were used to identify significant prognostic factors and reported as hazard ratios with corresponding 95% confidence intervals. Variables with *P* values lower than .05 in univariate Cox proportional hazard models were further evaluated in the multivariate Cox proportional hazard model. On the basis of the results of the multivariate Cox proportional hazard model, we constructed nomograms for 3‐ and 5‐year OS and CSS by using the rms package in R software, version 3.5.1 (http://www.r-project.org/). Surgery, chemotherapy, and radiotherapy were not included in the nomogram, as it would then be able to quantify prognosis for patients initially presenting to clinic, preoperatively after local and systemic evaluation. Concordance index (C‐index) ranging from 0.5 (a very poor model) to 1.0 (a perfect model) was used to assess the performance of nomograms. In general, nomograms with a C‐index greater than 0.7 show a good predictive ability.^15^ In addition, on the basis of bootstrap 1000 resampling validation, calibration curves were performed to compare the consistency between nomogram‐predicted and actual survival. A two‐sided *P*<.05 was defined as significant.

## 3 RESULTS

### 3.1 Patient characteristics

A total of 5243 patients with diagnoses of CS were registered in the SEER database from 1973 to 2016, of whom 935 (17.8%) had primary tumors in the pelvis. According to the aforementioned inclusion and exclusion criteria, 262 patients in 13 states in the United States, were eligible for and were eventually enrolled in this study (Figure 1).

Among these 262 patients, the median age was 52 years (range, 9‐88 years). As shown in Table 1, 161 (61.5%) were male and 101 (38.5%) were female. Of the whole population, the majority of patients was White (n = 225 [85.9%]), and 218 (83.2%) had conventional CS. Of the pathologic grades of disease, Grade II was the most common (n = 117 [44.6%]), followed by Grade I (n = 78 [29.8%]), Grade III (n = 49 [18.7%]), and Grade IV (n = 18 [6.9%]). In 90 patients (34.4%), the tumor size was less than 8 cm; in 98 (37.4%), the tumor size was between 8 and 13 cm; and in the 74 others (28.2%), the tumor size was more than 8 cm. Localized and regional were the most common tumor stages, accounting for 44.6% and 44.3%, respectively. In addition, surgery was performed in most patients (n = 220 [84.0%]), and chemotherapy and radiotherapy were performed in small numbers of patients (n = 33 [12.6%] and n = 18 [6.9%], respectively).

**Table 1:** Patient characteristics and 3‐ and 5‐year OS and CSS rates

				OS		CSS	
Characteristics	Number of patients		Percent	3‐year	5‐year	3‐year	5‐year
Age (years)							
<40		67	25.6	83.3%±5.1%	75.9%±6.2%	85.1%±4.9%	77.6%±6.1%
40‐59		117	44.6	79.7%±4.1%	74.5%±4.6%	79.7%±4.1%	75.7%±4.5%
≥60		78	29.8	58.2%±6.1%	45.9%±6.6%	62.8%±6.1%	57.6%±6.6%
Sex							
Male		161	61.5	69.1%±4.0%	60.5%±4.4%	72.1%±3.9%	65.8%±4.3%
Female		101	38.5	82.2%±4.2%	75.7%±5.0%	82.2%±4.2%	78.9%±4.7%
Race							
White		225	85.9	76.2%±3.1%	68.0%±3.5%	78.5%±3.0%	72.6 ± 3.4%
Others		37	14.1	59.3%±9.8%	54.4%±10.2%	59.3%±9.8%	59.3%±9.8%
Histologic subtype							
Conventional		218	83.2	76.8%±3.1%	69.6%±3.6%	78.1%±3.1%	74.3%±3.4%
Myxoid		17	6.5	83.3%±10.8%	66.7%±13.6%	83.3%±10.8%	66.7%±13.6%
Dedifferentiated		27	10.3	47.3%±10.4%	40.5%±10.9%	56.5%±10.0%	48.5%±11.4%
Pathologic grade							
Grade I		78	29.8	82.5%±4.6%	78.7%±5.1%	84.8%±4.5%	84.8%±4.5%
Grade II		117	44.6	80.2%±4.1%	73.7%±4.7%	81.2%±4.0%	77.0%±4.5%
Grade III		49	18.7	61.2%±7.9%	41.0%±9.2%	66.3%±7.7%	47.9%±9.7%
Grade IV		18	6.9	34.0%±11.9%	27.2%±11.3%	34.0%±11.9%	27.2%±11.3%
Tumor size (cm)							
＜8		90	34.4	83.0%±4.3%	76.7%±5.0%	78.1%±3.1%	74.3%±3.4%
8‐13		98	37.4	79.5%±4.4%	71.0%±5.3%	83.3%±10.8%	66.7%±13.6%
>13		74	28.2	57.1%±6.4%	47.8%±6.8%	56.5%±10.0%	48.5%±11.4%
Tumor stage							
Localized		117	44.6	87.5%±3.4%	80.7%±4.3%	89.3%±3.2%	87.9%±3.5%
Regional		116	44.3	74.2%±4.5%	63.5%±5.2%	76.7%±4.4%	66.8%±5.2%
Distant		29	11.1	19.4%±8.3%	19.4%±8.3%	19.4%±8.3%	19.4%±8.3%
Surgery							
No		42	16.0	40.9%±8.3%	40.9%±8.3%	43.8%±8.6%	43.8%±8.6%
Yes		220	84.0	80.4%±3.0%	71.0%±3.6%	81.9%±2.9%	75.6%±3.4%
Chemotherapy							
No		229	87.4	81.1%±2.8%	72.0%±3.5%	82.9%±2.7%	76.8%±3.3%
Yes		33	12.6	30.0%±8.6%	30.0%±8.6%	31.9%±9.0%	31.9%±9.0%
Radiotherapy							
No		244	93.1	74.4%±3.1%	67.9%±3.4%	76.0%±3.0%	71.2%±3.3%
Yes		18	6.9	69.6%±12.7%	45.1%±14.2%	76.0%±12.2%	63.3%±15.4%

Abbreviations: CSS, cancer‐specific survival; OS, overall survival.

### 3.2 Factors associated with OS

As shown in Table 2, univariate and multivariate analyses were conducted for OS. According to the univariate analysis, OS was significantly associated with age (*P* < .001), sex (*P* = .012), histologic subtype (*P* = .002), pathologic grade (*P* < .001), tumor size (*P* = .001), tumor stage (*P* < .001), surgery (*P* < .001), and chemotherapy (*P* < .001), whereas no significant differences were observed in OS with regard to race (*P* = .155) or radiotherapy (*P* = .187). Significant factors identified by univariate analysis were further explored in multivariate analysis, which showed that sex (*P* = .036), pathologic grade (*P* = .008), tumor size (*P* = .011), tumor stage (*P* = .003), and surgery (*P* = .001) were the independent risk factors.

**Table 2:** Univariate and multivariate Cox proportional hazards regression analyses of variables associated with overall survival

	Univariate analysis			Multivariable analysis		
Characteristics	HR	95% CI	*P* value	HR	95% CI	*P* value
Age (years)						
<40	Reference		<.001	Reference		.134
40‐59	1.464	0.768‐2.790	.247	1.020	0.519‐2.003	.955
≥60	3.346	1.776‐6.304	<.001	1.670	0.840‐3.322	.144
Sex						
Male	Reference		.012	Reference		.036
Female	0.535	0.328‐0.871	.012	0.576	0.344‐0.965	.036
Race						
White	Reference		.155	Not included		
Others	1.520	0.854‐2.706	.155			
Histologic subtype						
Conventional	Reference		.002	Reference		.257
Myxoid	0.919	0.370‐2.285	.855	0.626	0.238‐1.643	.341
Dedifferentiated	2.774	1.549‐4.965	.001	1.607	0.797‐3.240	.185
Pathologic grade						
Grade I	Reference		<.001	Reference		.008
Grade II	1.103	0.604‐2.016	.749	0.928	0.481‐1.791	.824
Grade III	3.459	1.850‐6.467	<.001	2.245	1.097‐4.593	.027
Grade IV	5.335	2.584‐11.015	<.001	2.988	1.135‐7.871	.027
Tumor size (cm)						
＜8	Reference		.001	Reference		.011
8‐13	1.417	0.794‐2.527	.238	1.051	0.575‐1.923	.871
＞13	2.796	1.612‐4.849	<.001	2.151	1.189‐3.892	.011
Tumor stage						
Localized	Reference		<.001	Reference		.003
Regional	2.110	1.241‐3.587	.006	1.289	0.725‐2.292	.388
Distant	9.460	5.159‐17.347	<.001	4.034	1.741‐9.349	.001
Surgery						
No	Reference		<.001	Reference		.001
Yes	0.338	0.208‐0.548	<.001	0.380	0.213‐0.680	.001
Chemotherapy						
No	Reference		<.001	Reference		.669
Yes	3.651	2.249‐5.927	<.001	1.156	0.594‐2.249	.669
Radiotherapy						
No	Reference		.187	Not included		
Yes	1.638	0.787‐3.407	.187			

### 3.3 Factors associated with CSS

As shown in Table 3, univariate analysis indicated that age (*P* = .003), sex (*P* = .042), histologic subtype (*P* = .007), pathologic grade (*P* < .001), tumor size (*P* = .001), tumor stage (*P* < .001), surgery (*P* < .001), and chemotherapy (*P* < .001) were significantly associated with CSS, whereas race (*P* = .202) and radiotherapy (*P* = .776) were not significantly correlated with CSS. The multivariate analysis identified pathologic grade (*P* = .005), tumor size (*P* = .016), tumor stage (*P* = .001), and surgery (*P* = .002) as the independent risk factors in CSS.

**Table 3:** Univariate and multivariate Cox proportional hazards regression analyses of variables associated with cancer‐specific survival

	Univariate analysis			Multivariable analysis		
Characteristics	HR	95% CI	*P* value	HR	95% CI	*P* value
Age (years)						
<40	Reference		.003	Reference		.636
40‐59	1.429	0.729‐2.801	.298	0.978	0.481‐1.992	.952
≥60	2.805	1.429‐5.505	.003	1.277	0.611‐2.670	.515
Sex						
Male	Reference		.042	Reference		.129
Female	0.584	0.348‐0.981	.042	0.651	0.374‐1.132	.129
Race						
White	Reference		.202	Not included		
Others	1.500	0.805‐2.798	.202			
Histologic subtype						
Conventional	Reference		.007	Reference		.530
Myxoid	1.099	0.439‐2.751	.840	0.710	0.269‐1.875	.489
Dedifferentiated	2.767	1.474‐5.195	.002	1.412	0.657‐3.035	.377
Pathologic grade						
Grade I	Reference		<.001	Reference		.005
Grade II	1.511	0.744‐3.071	0.254	1.151	0.535‐2.475	.719
Grade III	4.576	2.197‐9.531	<.001	2.773	1.221‐6.298	.015
Grade IV	7.992	3.571‐17.884	<.001	4.225	1.465‐12.183	.008
Tumor size (cm)						
＜8	Reference		.001	Reference		.016
8‐13	1.509	0.797‐2.857	.207	1.062	0.540‐2.090	.862
＞13	3.055	1.669‐5.594	<.001	2.221	1.159‐4.256	.016
Tumor stage						
Localized	Reference		<.001	Reference		.001
Regional	2.758	1.480‐5.141	.001	1.693	0.871‐3.289	.120
Distant	13.608	6.911‐26.793	<.001	5.596	2.245‐13.953	<.001
Surgery						
No	Reference		<.001	Reference		.002
Yes	0.329	0.195‐0.554	<.001	0.357	0.189‐0.674	.002
Chemotherapy						
No	Reference		<.001	Reference		.815
Yes	4.203	2.531‐6.981	<.001	1.088	0.537‐2.203	.815
Radiotherapy						
No	Reference		.776	Not included		
Yes	1.141	0.459‐2.841	.776			

### 3.4 Kaplan‐Meier curve analyses

**Figure 2:**
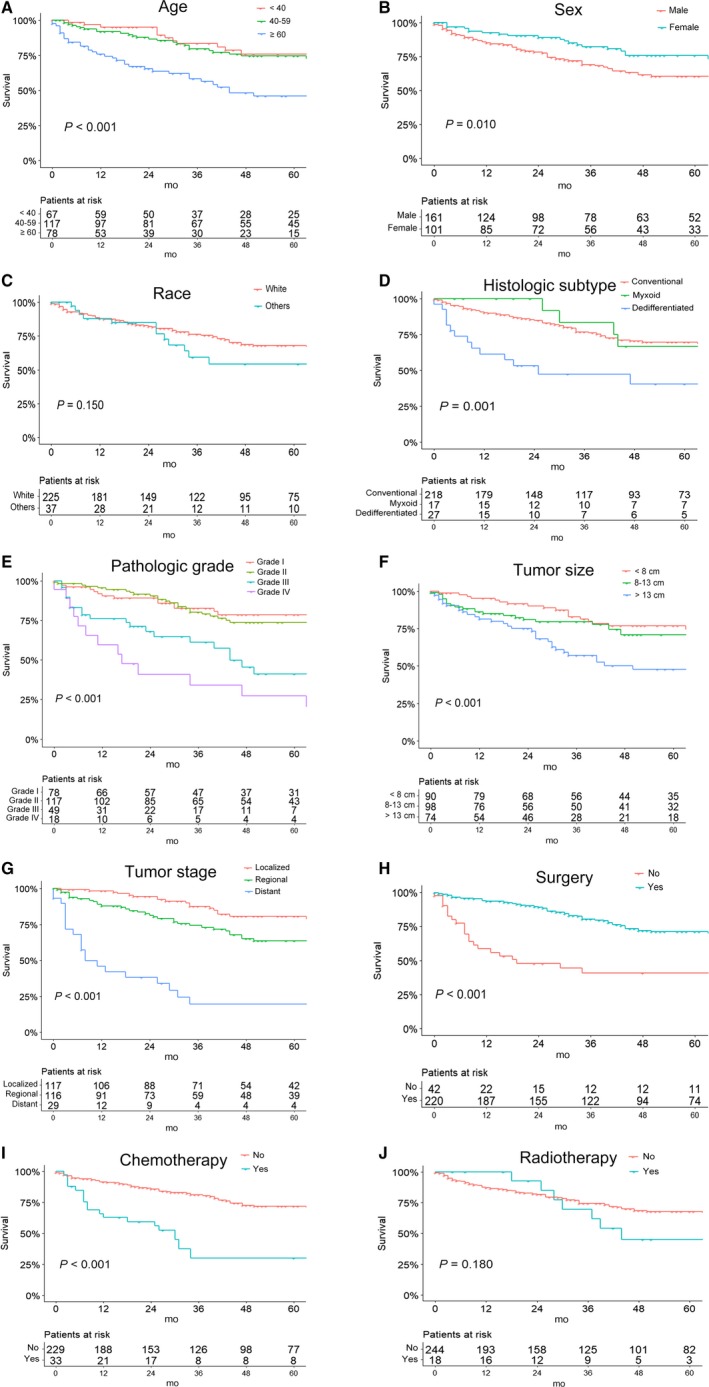
Kaplan‐Meier curves of overall survival for patients based on (A) age, (B) sex, (C) race, (D) histologic subtype, (E) pathologic grade, (F) tumor size, (G) tumor stage, (H) use of surgery, (I) use of chemotherapy, and (J) use of radiotherapy

**Figure 3:**
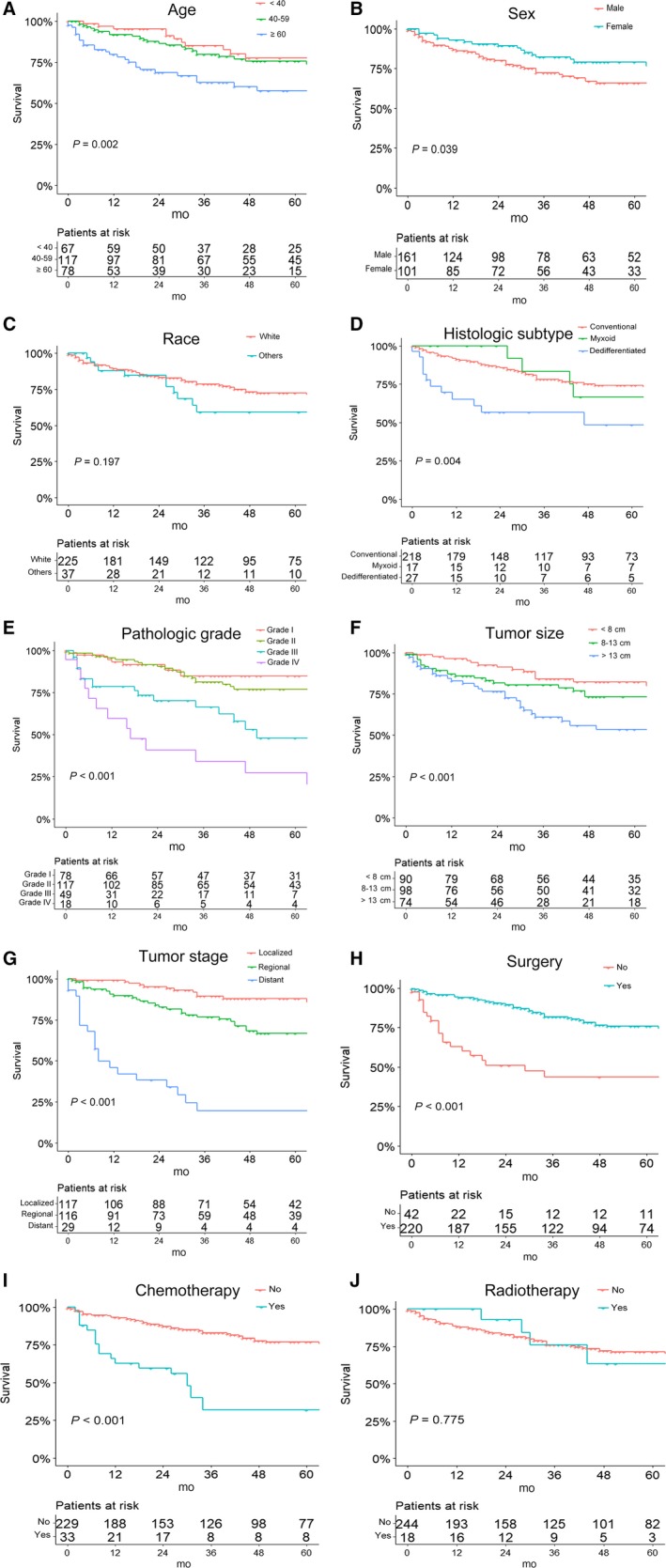
Kaplan‐Meier survival curves of cancer‐specific survival for patients based on (A) age, (B) sex, (C) race, (D) histologic subtype, (E) pathologic grade, (F) tumor size, (G) tumor stage, (H) use of surgery, (I) use of chemotherapy, and (J) use of radiotherapy

According to the Kaplan‐Meier curves and log‐rank analyses of OS and CSS (Figures 2 and 3), younger patients and female patients had a better prognosis. Moreover, tumors of lower pathologic grade, smaller size, and localized stage were associated with a better outcome. Patients in whom the histologic subtype of CS was dedifferentiated had worse rates of survival than did those with the other two histologic subtypes. In addition, patients who underwent surgery had better survival rates than those who did not have surgical treatment. Conversely, survival rates were worse among patients who underwent chemotherapy. No significant differences were observed in survival with regard to race and radiotherapy.

### 3.5 Predictive nomogram

**Figure 4:**
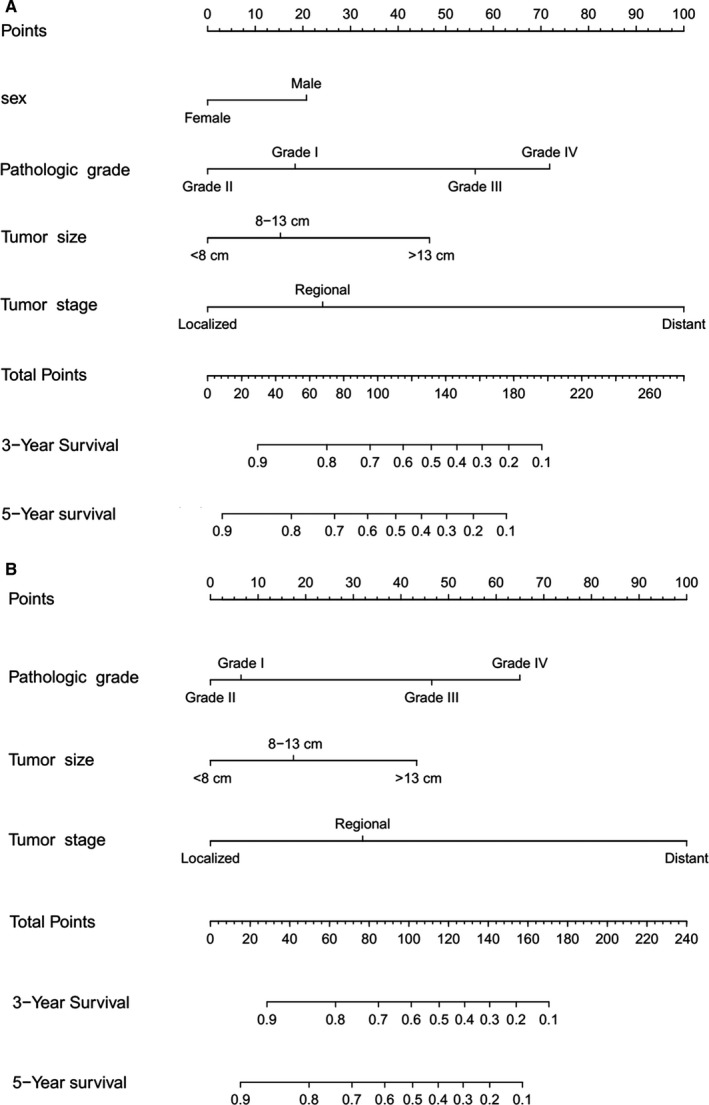
Nomogram to predict overall survival and cancer‐specific survival (CSS) in patients with pelvic chondrosarcoma. A, Predicting 3‐year and 5‐year overall survival rates, (B) Predicting 3‐year and 5‐year CSS rates

**Figure 5:**
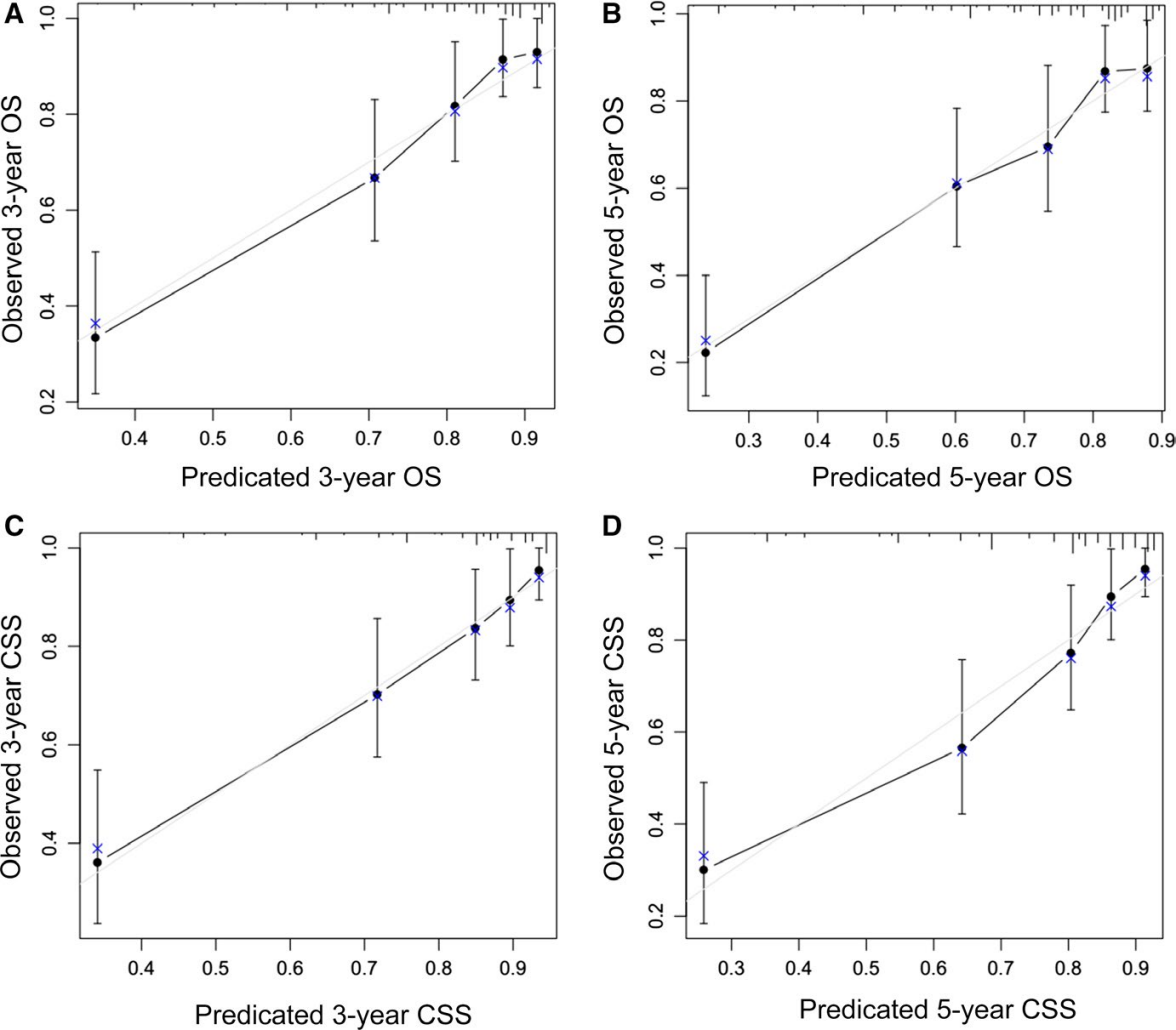
Calibration curves of the nomogram predicting overall survival and cancer‐specific survival (CSS) in patients with pelvic chondrosarcoma. A, 3‐year overall survival rate, (B) 5‐year overall survival rate, (C) 3‐year CSS rate, and (D) 5‐year CSS rate

The significantly independent risk factors identified by multivariate analyses, except surgery were integrated to construct the prognostic nomograms for predicting 3‐ and 5‐year OS and CSS of patients with pelvic CS (Figure 4). The point scale at the top of each nomograms was used first to give every prognostic variable a score; then the scale at the bottom of each nomogram was used (adding up the scores of all variables) to predict the 3‐ and 5‐year survival rates. The nomogram discrimination for OS prediction revealed that tumor stage contributed most to prognosis, followed by pathologic grade of the tumor, tumor size, and sex. With regard to CSS, nomograms showed that tumor stage was also the most important factor affecting outcome, followed by pathologic grade and tumor size. In addition, internal validation for nomograms was performed by C‐index and calibration. C‐index values of nomogram predictions of OS and CSS were 0.758 and 0.786, respectively, which suggest that these models made accurate predictions. Through the calibration curves (Figure 5), nomogram prediction proved to have excellent agreement with actual survival.

## 4 DISCUSSION

A variety of prognostic factors influences the survival outcome of patients with cancer, and the ability of a single prognostic factor to predict individual survival probability is limited. In addition, relying merely on traditional staging systems is not enough to accurately assess cancer prognosis.^16^ Because of the ability to visually show data, accuracy, and individualization, nomograms have been commonly used to predict the survival of patients with cancer.^17^ However, to date, nomograms predicting survival specifically of patients with pelvic CS had not yet been reported. Thus, we sought to develop and validate comprehensive nomograms for OS and CSS of patients with pelvic CS on the basis of data from 262 cases in the SEER database, which covers 28% of the US population. As a result, nomograms for 3‐ and 5‐year OS and CSS were developed and their prediction performance was validated, which could promote the popularization of personalized treatment and survival evaluation.

Of the eligible patients, the majority was older than 40 years, and prognosis for survival worsened with the age, according to the Kaplan‐Meier curves and log‐rank analyses. Interestingly, this difference was found not to be independent in the multivariate analysis, which was a finding similar to those in previous studies.^18^, ^19^, ^20^ At present, the role of sex in CS is still controversial. Some previous studies showed that sex was an independent risk factor, while another several studies revealed that sex was not a prognostic factor.^21^, ^22^, ^23^, ^24^ In this study, the ratio of male to female patients was ~1.6:1, which was consistent with such ratios in previous studies.^23^, ^25^ Being female was associated with a better prognosis; thus sex was an independent prognostic factor with obvious effects on OS of patients with pelvic CS. Overall, the findings of this study provided further evidence in terms of the relationship between demographic characteristics and prognosis.

In general, pathological grade plays an important role in the prognosis of patients with cancer. Studies showed that patients with Grade I pelvic CS had better rates of survival; the 5‐year survival rate reached 90% among patients with the lowest degree of malignancy and the lowest possibility of distant metastasis. Grades II and III pelvic CS tend to metastasize in the early stages, and the 5‐year survival rate was about 40% to 50% in patients with these grades of tumors. Patients with grade IV pelvic CS had the worst prognosis, with a 5‐year survival rate of 10%‐20%.^26^, ^27^ In our research, we found similar 5‐year survival rates for each pathological grade, which confirmed the great contribution of pathological grade to prognosis of CS in the pelvis, which was consistent with that of CS in other sites. Furthermore, the higher the pathological grade of the tumor is, the more likely it is to recur, which increases the risk of death.^28^ Higher pathological grade is correlated with the development of distant metastasis, which also worsens the prognosis for survival, and tumor stage was identified as another important independent risk factor. Such a trend further demonstrates the importance of early diagnosis, inasmuch as detection of CS originating in the pelvis is prone to delay because the early symptoms are vague.^29^

Although considerable progress has been made in the treatment of sarcomas, the prognosis of patients with primary pelvic sarcomas is still poorer than that of sarcomas at other sites. Accordingly, there is much controversy about optimal surgical treatment, systemic chemotherapy, and radiotherapy. In congruence with previous studies, we found that surgery confers a significant advantage in OS and CSS of patients with pelvic CS.^30^, ^31^ Of the types of surgery involved, wide surgical resection was a significant prognostic factor for the long‐term survival and local recurrence of decrease, and individual reconstruction was a key to maintaining good function.^6^ However, with regard to nonoperative treatment, both chemotherapy and radiotherapy have limited effects on improving the prognosis of patients with pelvic CS. A possible explanation for this is that CS is composed of abundant extracellular matrix with poor vascularity, which could result in its primary resistance to chemotherapy and radiotherapy.^32^ In addition, the expression of P‐glycoprotein in CS is one of the important mechanisms of resistance to chemotherapy. Resistance to radiotherapy may be attributed to loss of tumor suppressor p16 in CS and to an increase in the expression of antiapoptotic proteins Bcl‐2, Bcl‐xL, and X‐linked inhibitor of apoptosis protein.^33^

There were some limitations of this study that should be noted. First, the nomograms were developed on the basis of retrospective data. Although the SEER database represents 28% of the US population, it is inevitable that certain patient information was insufficient, and so larger prospective studies could further validate the reliability of the nomograms. Second, because some clinicopathological parameters such as surgical margin status and local recurrence were unavailable in the SEER database, such variables were not included in this study. Besides, there was no discrimination between the type of surgery performed, as well as specific chemotherapy regimen and detailed information regarding patients with syndromic conditions could not be provided. Third, pathological grade in SEER database was classified into four categories, while CS was graded from I to III according to WHO classification, which may be somewhat inaccurate. Dedifferentiated and myxoid CS were not found to be risk factors as well, which may be due to the limited numbers in this series, while they were known to be associated with a poorer prognosis. Finally, although C‐index is a good tool for validating nomograms, the nomograms could be more reliable if external validations were performed with another independent large‐scale dataset.

## 5 CONCLUSION

With data from a large population‐based cohort, we developed and validated nomograms to provide individualized estimates of rates of 3‐ and 5‐year OS and CSS in patients with pelvic CS for the first time. The nomograms showed good performance in accuracy and applicability. Therefore, we strongly recommend applying these nomograms in personalized prognostic evaluation of patients with pelvic CS.

## CONFLICT OF INTEREST

The authors declare that they have no conflict of interest.
